# Ophthalmological Microvascular Changes in ANOCA/INOCA Disease and Ophthalmological Methods to Detect Them—A Systematic Review

**DOI:** 10.3390/jcm15041344

**Published:** 2026-02-08

**Authors:** Małgorzata Ryk-Adamska, Maciej Janiszewski, Mariusz Tomaniak, Jacek Pawel Szaflik, Przemysław Kasiak, Anna Zaleska-Żmijewska

**Affiliations:** 1 Department of Ophthalmology, Public Ophthalmic Clinical Hospital (SPKSO), Medical University of Warsaw, 02-015 Warsaw, Poland; 2 Public Ophthalmic Clinical Hospital (SPKSO), 03-709 Warsaw, Poland; 3 Department of Heart Failure and Cardiac Rehabilitation, Medical University of Warsaw, ul. Kondratowicza 8, 03-242 Warsaw, Poland; 4 First Department of Cardiology, Medical University of Warsaw, ul. Banacha 1A, 02-097 Warsaw, Poland; 5 Central Clinical Hospital—University Clinical Centre, Medical University of Warsaw, ul. Banacha 1A, 02-091 Warsaw, Poland

**Keywords:** non-obstructive coronary artery disease, angina with non-obstructive coronary arteries, ischemia with non-obstructive coronary arteries, microvascular angina, coronary microvascular dysfunction, retina, choroid, conjunctiva

## Abstract

**Background/Objectives:** Coronary artery disease (CAD) remains one of the leading cardiovascular diseases worldwide. While obstructive CAD is well characterized and managed, identification of patients with non-obstructive CAD (NOCAD) remains challenging. Unlike the coronary vasculature, the eye’s microcirculation can be easily and non-invasively assessed. Therefore, this systematic review summarized the ophthalmological diagnostic methods used to assess microvascular alterations associated with coronary microvascular dysfunction (CMD), angina with non-obstructive coronary arteries (ANOCA), or ischemia with non-obstructive coronary arteries (INOCA). **Methods:** According to PRISMA guidelines, PubMed/MEDLINE and Embase databases were screened by two independent reviewers from inception to 25 November 2025. Original articles that examined ophthalmological microvascular changes by any method in adults with CMD or its subtypes were included. The quality of the studies was assessed using the JBI Critical Appraisal Checklist. **Results:** Of 101 identified articles, nine studies met the inclusion criteria, comprising 1894 patients. Optical coherence tomography angiography was the most frequently used imaging modality, followed by optical coherence tomography, slit-lamp smartphone imaging, and fundus photography. Five investigations employed blinded image analysis, three did not, and one study used it partially. Four studies used semi-automated measurements, four employed fully automated methods, and one study applied manual and automated measurements for different parameters. **Conclusions:** Despite a limited number of studies, retinal and conjunctival microvascular alterations helped differentiate CAD subtypes and may reflect systemic microcirculatory impairment among patients with ANOCA/INOCA. Ophthalmological imaging techniques have the potential to serve as non-invasive tools for detecting microvascular alterations associated with CMD in ANOCA and INOCA patients. PROSPERO Registration Number: CRD420251239875

## 1. Introduction

Despite continuous advances in prevention, diagnostics, and therapy over recent decades, coronary artery disease (CAD) remains a leading cause of morbidity and mortality worldwide [1]. The traditional diagnosis of CAD is based on typical clinical symptoms, predominantly chest pain, evidence of myocardial ischemia on stress testing, and the presence of significant coronary artery stenoses demonstrated by coronary computed tomography angiography (CCTA) or invasive coronary angiography (ICA). However, in clinical practice, a substantial proportion of patients presenting with anginal symptoms or evidence of myocardial ischemia on functional testing do not exhibit such obstructive lesions in the coronary arteries [2].

The term non-obstructive coronary artery disease (NOCAD) is the broadest concept and refers to the presence of symptoms suggestive of myocardial ischemia, with or without accompanying ischemic changes in additional diagnostic tests, in patients without significant stenoses in the epicardial coronary arteries. One of the most important underlying causes of NOCAD is coronary microvascular dysfunction (CMD). According to the most recent European Society of Cardiology (ESC) Guidelines from 2024, NOCAD patients suffering from angina or its equivalents should be stratified as angina with non-obstructive coronary arteries (ANOCA) or ischemia with non-obstructive coronary arteries (INOCA) when myocardial ischemia is documented. The term microvascular angina (MVA) represents one of the two major endotypes of ANOCA/INOCA, alongside vasospastic angina (VSA) [2].

The exact prevalence of ANOCA/INOCA remains challenging to quantify due to heterogeneity in diagnostic criteria and under-recognition in clinical practice. It is estimated that up to 60% of women and 30% of men referred for ICA due to symptoms suggestive of CAD have no significant atherosclerotic narrowing in the epicardial coronary arteries [2]. Historically, ANOCA and INOCA were often considered benign or predominantly functional conditions, leading to their underdiagnosis and marginalization in clinical guidelines. However, an increasing body of evidence indicates that patients with ANOCA/INOCA are at elevated risk of major adverse cardiovascular events, including myocardial infarction, heart failure, and cardiac death. Moreover, these patients frequently experience substantially impaired quality of life [3,4,5].

The first step in diagnosis is estimating the pre-test likelihood of obstructive CAD (OCAD) with the risk-factor-weighted clinical likelihood (RF-CL) model to guide diagnostic testing and avoid unnecessary procedures in low-risk patients. Although RF-CL demonstrates higher sensitivity than previous models in identifying patients with a very low likelihood (≤5%), particularly when combined with the coronary artery calcium score, there is currently no validated model capable of estimating the probability of ANOCA/INOCA [2,6].

The diagnostic workup of ANOCA/INOCA remains complex, requiring a multimodal approach [2]. As CMD may arise from distinct pathophysiological mechanisms, current evidence supports the use of multiple, mechanism-oriented diagnostic pathways integrating invasive and non-invasive tests [7]. The availability of non-invasive techniques remains limited and insufficient to meet the clinical demand arising from the growing prevalence of ANOCA/INOCA. Moreover, the invasive techniques are associated with a risk of potential adverse events, necessitating careful patient selection [2]. Consequently, there is an ongoing search for simple, non-invasive, and widely applicable diagnostic methods that would enable the identification of patients with ANOCA/INOCA.

The prognosis of patients with ANOCA/INOCA depends on the underlying pathophysiological mechanism and treatment effectiveness. Management focuses on cardiovascular risk-factor control, lifestyle modification, strict control of comorbidities (e.g., diabetes, dyslipidaemia), avoidance of vasospasm-inducing substances, and endotype-specific pharmacotherapy [2].

There is growing interest in ophthalmological possibilities for assessing the microcirculation of the eye as a window into coronary microcirculation. In contrast to the coronary vasculature, the ocular microcirculation can be easily and non-invasively evaluated due to its accessible location [8,9]. This provides a unique opportunity to directly assess microcirculation in vivo, in real time, and repeatedly, without the need for invasive procedures. Retinal arterioles share several key physiological and anatomical features with coronary microvessels [8]. Additionally, both retinal and coronary microcirculation are influenced by the same major cardiovascular risk factors, including hypertension, diabetes, and dyslipidemia [10,11,12]. Therefore, changes in the retinal microvasculature may serve as non-invasive indicators of systemic microvascular dysfunction relevant to coronary disease [8]. The potential clinical relevance of retinal microvascular changes has been widely investigated. Previous studies have found that a range of retinal microvascular alterations, such as generalized and focal arteriolar narrowing, arteriovenous nicking, and retinopathy, may serve as markers for cardiovascular disease [8]. Moreover, retinal imaging has been proposed as a non-invasive tool for cardiovascular risk stratification [13,14].

Previous reviews primarily concentrate on OCAD and are often limited to one specific imaging modality. While prior reviews have occasionally included ANOCA/INOCA patients as part of broader study groups, they have not made this subgroup a central focus, and data specific to these patients are often not extractable. Numerous studies have been conducted to establish the correlation between retinal changes and CAD; however, few have examined microvascular alterations in patients with ANOCA/INOCA disease [9,15].

The patients with NOCAD, including those with ANOCA/INOCA, are often underdiagnosed and clinically challenging. Therefore, this systematic review aimed to investigate the potential of ophthalmological diagnostic methods in identifying patients with ANOCA/INOCA disease.

## 2. Materials and Methods

### 2.1. Searching Strategy

This systematic review was conducted in accordance with the PRISMA guidelines [16] (PRISMA checklist in Supplementary Materials, Table S1). PubMed/MEDLINE and Embase databases were searched from their inception up to 25 November 2025. The protocol was registered in PROSPERO (ID: CRD420251239875). The following searching strategy was used for both databases: (“ischaemia with non-obstructive coronary arteries” OR “angina with non-obstructive coronary arteries” OR “coronary microvascular dysfunction” OR “microvascular angina” OR “microvascular coronary disease” OR “cardiac syndrome X” OR “myocardial infarction with non-obstructive coronary arteries” OR “non-obstructive coronary artery disease” OR “normal coronary angiography” OR “normal angiographic”) AND (“eye” OR “retina” OR “choroid” OR “retinal vessels” OR “retinal microvasculature” OR “retinal microcirculation” OR “chorioretinal microvasculature” OR “choroidal microvasculature” OR “conjuntival microvasculature”).

### 2.2. Inclusion and Exclusion Criteria

Inclusion criteria were determined based on PICOS principles (population, intervention, comparison, outcome, study type). Therefore, the population was defined as adults (≥18 years old) with NOCAD or its subtypes, as explicitly defined by the authors or as identifiable based on the cardiological criteria provided. Eligible patients were those who had undergone ICA or CCTA to rule out obstructive coronary disease and who presented with angina symptoms, angina equivalents, and/or objective evidence of myocardial ischemia on non-invasive testing. Intervention was defined as any kind of ophthalmologic assessment of the microvasculature (optical coherence tomography angiography (OCTA), slit-lamp smartphone imaging, fundus photography, etc.). Comparison was defined as healthy controls, OCAD, or other NOCAD subtypes. Studies without control groups were also considered if they met other inclusion criteria. Outcome was defined as any available result of microvascular assessments (e.g., vessel density (VD), choroidal thickness, etc.). Only original articles were included. All other publication types, such as reviews, editorials, letters to the editor, short communications, conference abstracts, etc., were not considered. There were no language limitations. As the terminology used to describe ischemia and angina in the absence of obstructive coronary artery disease has evolved, studies have used a range of historical terms. The ANOCA and INOCA concepts were formalized only recently and were not uniformly applied in earlier literature. To ensure consistent classification, all cohorts were mapped to ANOCA/INOCA phenotypes whenever sufficient clinical information was available.

Although our primary focus was chronic non-obstructive ischemic syndromes (ANOCA/INOCA), we also permitted inclusion of studies enrolling patients with myocardial infarction with non-obstructive coronary arteries (MINOCA), provided that obstructive epicardial CAD was excluded and ocular microvascular assessment was performed. MINOCA was treated as a distinct clinical entity, and its findings were described separately to avoid conflating post-infarction mechanisms with chronic ischemic syndromes.

Exclusion criteria were determined as follows: studies in which NOCAD patients were part of a broader population and their data could not be analyzed separately, studies in which NOCAD was defined solely by angiographic stenosis severity (e.g., Gensini score thresholds) without accompanying clinical criteria (angina or documented ischemia) or without any diagnostic evaluation of coronary microvascular function, studies in which coronary anatomy was not confirmed by ICA or CCTA, and studies that did not report any ophthalmologic microvascular outcomes. In addition, studies were excluded if the coronary slow-flow phenomenon (CSFP) was used as the sole criterion for defining microvascular dysfunction in the absence of mechanism-oriented diagnostic testing.

### 2.3. Used Terminology

In this review, we use ANOCA/INOCA to describe patients with angina and/or objective ischemia with non-obstructive epicardial coronary arteries, in line with contemporary ESC nomenclature. We use NOCAD only as a broader descriptive umbrella term to reflect the wording of some original studies and/or the overarching concept of non-obstructive epicardial CAD. When applicable, study populations were reclassified into ANOCA/INOCA (and related subgroups such as CMD) based on the information provided by the authors.

### 2.4. Screening and Data Extraction

Firstly, the identified records were uploaded into EndNote (version 21.5; Clarivate Analytics, Philadelphia, PA, USA), and duplicates were removed. Then, two authors (MRA and PK) independently screened the title and abstract. Subsequently, those two authors independently extracted the data from the included articles. The extracted data were study type, usage of blinded image analysis, measurement method, imaging method, characteristics of the NOCAD subgroup, characteristics of the comparison group, number of participants (if available, performed separately for NOCAD subgroup and comparison group), analyzed parameters, key findings, and confounders (if available, stratified for only measured, measured and statistically adjusted, and excluded). Any discrepancies were resolved through the discussion.

### 2.5. Risk of Bias Assessment

All identified articles appear to be cross-sectional studies. Therefore, the JBI Critical Appraisal Checklist for Analytical Cross-Sectional Studies was used [17]. Two reviewers (MRA and PK) independently graded the selected articles in an 8-item list. Each item was graded as Yes, No, Unclear, or Not Applicable. Discrepancies were resolved through discussion.

### 2.6. Use of Artificial Intelligence Tools

ChatGPT (OpenAI, GPT-5.2) was used to assist with English language editing of the manuscript.

## 3. Results

### 3.1. Characteristics of Included Studies

**Figure 1:**
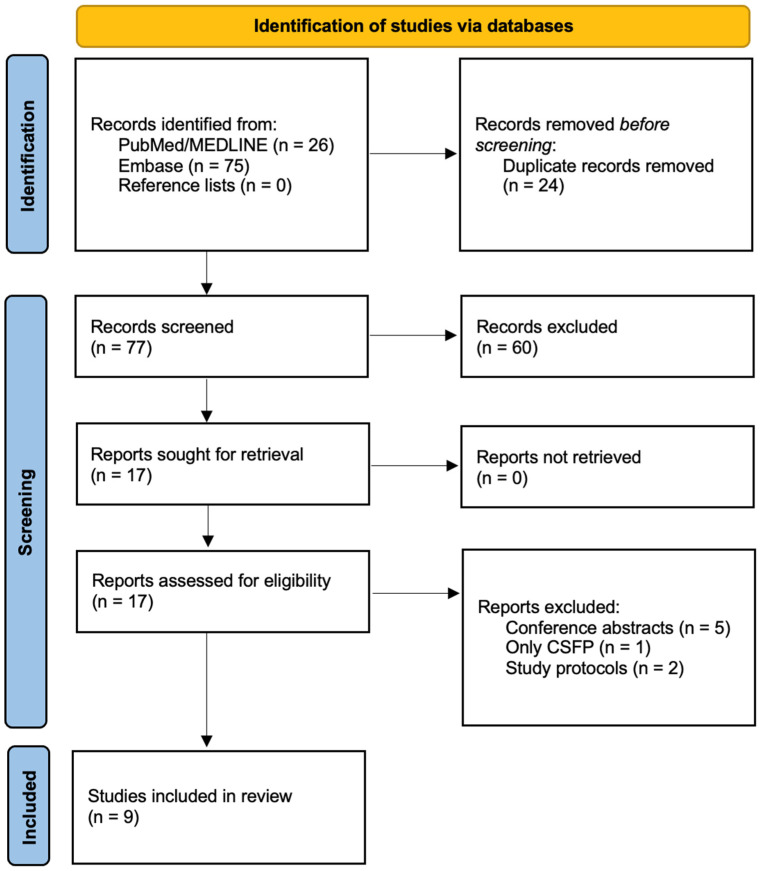
Flowchart for screening protocol. Abbreviations: CSFP, coronary slow-flow phenomenon.

Initially, 26 articles were identified in PubMed/MEDLINE and 75 articles in Embase. Of 101 articles, 24 were duplicates. Of 77 articles, 60 were excluded after screening their title and abstracts. Then, five conference abstracts [18,19,20,21,22], two study protocols [23,24], and one investigation with CSFP [25] were excluded. A precise screening protocol is presented in Figure 1.

Finally, nine articles were included. Studies vary in the characteristics of their study groups. Three studies defined the study group as MVA [26,27,28], two as NOCAD [29,30], three as INOCA [31,32,33], and one as a normal angiographic patient with typical chest pain or anginal equivalents [34]. Moreover, the definition of NOCAD/MVA/ANOCA/INOCA subgroups varies across studies. Liew et al. [26] defined MVA as chest pain with less than 25% stenosis in all coronary epicardial arteries, whereas in the study by Xu et al. [27], MVA patients had either normal coronary angiography or stenosis < 50% in primary branches and <75% in secondary branches. In a study by Kanar et al. [28], patients with MVA were reported to have normal epicardial arteries, but no information was provided regarding the degree of stenosis. In Table 1, the study groups were reclassified according to the new nomenclature regarding the 2024 ESC Guidelines for the management of chronic coronary syndromes and presented with the original terminology used in the individual studies.

In all studies, adult patients presenting with cardiovascular symptoms underwent ICA to rule out the presence of OCAD, except for one study [29], where CCTA was used. Additionally, two patients with CMD assessed by positron emission tomography were included in Shiromani et al.’s study [32]. Nearly all studies involve comparisons between ANOCA/INOCA and other groups (e.g., OCAD, other subgroups of NOCAD, or healthy controls). In only one investigation [34] is there no dedicated control group, and this study compares its findings to normative values for normal populations.

Various ophthalmological imaging methods were used to assess microcirculation in the eye. The most common methods were OCTA (four studies), followed by spectral-domain optical coherence tomography (SD-OCT) (two studies), slit-lamp and smartphone imaging (two studies), and fundus photography (one study).

### 3.2. Quality of Included Studies

Qualitative assessment of the included studies is presented in Table 2. All studies were assessed using the JBI Critical Appraisal Tool, and a final decision was made on their inclusion. The inclusion criteria and study settings were clearly explained in the selected articles. Moreover, the objective criteria were used for measurements, and outcomes were measured reliably. Imaging and analysis protocols were generally reported in sufficient detail to ensure reproducibility. Moreover, several studies applied masked assessment and one study dual-observer evaluation with inter-observer agreement procedures, reducing operator-dependent bias and strengthening the reliability of outcome measurements. There were no studies with inappropriate statistical methods used. The majority of the included studies identified the confounding factors and coped well with them. However, Xu et al. [27], Agca et al. [31], and Eslami et al. [34] did not clearly describe strategies to address potential confounders. In these studies, confounders were listed in baseline characteristics, but the statistical analyses relied mainly on univariate comparisons, without multivariable adjustment, despite only partial control provided by group matching and exclusion criteria.

### 3.3. Results of Included Studies

Four studies assessed retinal microvascular parameters using OCTA and reported retinal microvascular alterations in ANOCA/INOCA populations. VD in the superficial capillary plexus (SCP) was assessed in all four studies. In two studies, VD in the SCP was reduced when NOCAD/INOCA patients were compared with healthy controls [29,31] and in one study when women with INOCA and CMD were compared with those without CMD [32]. Among three studies assessing VD in the deep capillary plexus (DCP), two reported a decrease in NOCAD/INOCA patients when compared with healthy controls [29,31]. One study found that 45% of normal angiographic patients had abnormal VD in SCP, and 8,3% had abnormal VD in DCP [34]. In one study, women with INOCA and CMD showed lower perfusion density in SCP when compared with women without CMD [32].

Findings for the peripapillary circulation were inconsistent. One study reported that VD in the radial peripapillary capillary (RPC) network did not differ between NOCAD and healthy controls, with reductions observed only in OCAD [29], whereas another study found reduced VD in RPC in both INOCA and OCAD compared with healthy controls [31].

Foveal avascular zone (FAZ) parameters were evaluated in three studies and showed no significant differences [31,32,34]. In one study, the foveal density in a 300 μm region around the FAZ (FD 300) was reduced in NOCAD compared to healthy controls, while retinal nerve fiber layer (RNFL) thickness did not differ between NOCAD and healthy controls [29].

Based on single studies, SD-OCT retinal vessel caliber parameters assessed with the full-width half-maximum (FWHM) method (retinal arterial lumen diameter (RALD), retinal arterial outer diameter (RAOD), arteriovenous ratio (AVR)) differentiated MVA from ischemic heart disease and healthy controls [27], whereas structural OCT parameters (subfoveal choroidal thickness (SFCT) and peripapillary retinal nerve fiber layer thickness (pRNFLT)) showed no discriminatory value for MVA [28].

In both studies evaluating conjunctival microvasculature, all examined flow parameters (axial velocity, cross-sectional velocity, and blood flow rate) differed significantly between the comparison groups (INOCA with CMD vs. control and NOCAD vs. MI), and the greatest alterations were most pronounced in smaller vessels [30,33].

One included study enrolled a MINOCA cohort; therefore, we report it as a separate subgroup from the chronic ANOCA/INOCA/NOCAD cohorts. In the single study using fundus photography, in women, a smaller retinal venular caliber was more frequently observed in MVA than in OCAD [26].

## 4. Discussion

To our knowledge, this is the first systematic review that attempts to gather findings from ophthalmological imaging in patients with ANOCA/INOCA. Only a few studies have specifically investigated ocular vascular changes in this group. This reflects the novelty of the topic and the fact that reliable diagnostic methods confirming the presence of CMD have only recently become available. The key insights are as follows. Firstly, ophthalmological imaging techniques show emerging potential for the non-invasive assessment of microvascular alterations in patients with ANOCA/INOCA. Secondly, the observed ocular microvascular changes are consistent with the concept that CMD is a part of a broader systemic microvascular disorder. Thirdly, current studies were conducted on relatively small cohorts, and further investigations are required to validate these observations.

**Figure 2:**
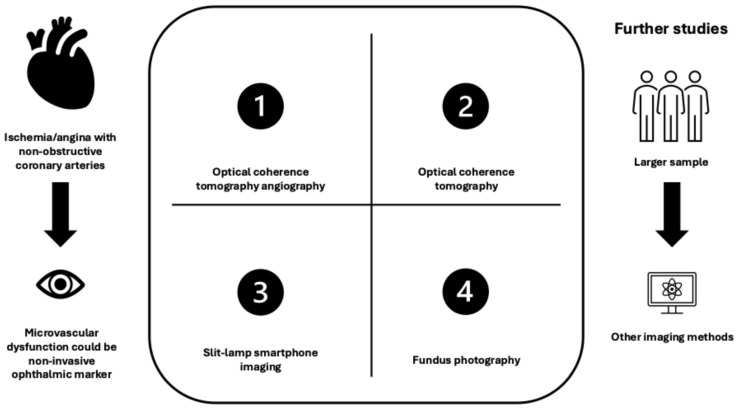
Comparison of ocular imaging modalities and their relevance to coronary microvascular dysfunction.

Figure 2 presents a comparison of ocular imaging modalities and their relevance to coronary microvascular dysfunction.

Patients with ANOCA/INOCA represent a challenging and often overlooked group. Although these patients do not have significant coronary artery stenosis, they remain at elevated risk for adverse cardiovascular events, including myocardial infarction and heart failure. Quality of life is often significantly impaired due to persistent anginal symptoms that can be challenging to manage [2]. The complicated diagnostic pathway in patients with ANOCA/INOCA often leads to multiple hospitalizations, repeated invasive procedures such as coronary angiography, and high healthcare costs [2,35]. Therefore, there is an urgent need for new diagnostic tools that are ideally non-invasive, repeatable, and easily performed on an outpatient basis. Hence, cardiologists have a significant interest in collaborating with ophthalmologists and analyzing microcirculatory disorders in the eye in the course of various cardiovascular diseases.

Among all described studies, the study group investigated by Shiromani et al. [32] and Mailey et al. [33] were the best characterized. Unlike other studies, they assessed patients for CMD as the underlying mechanism of ANOCA/INOCA. These patients underwent a complete diagnostic assessment, including both anatomical evaluation by ICA (similarly to other studies) and additionally functional testing of the coronary microcirculation. This dual approach allowed for the precise identification of patients with CMD, resulting in a more homogeneous study population. This approach was not utilized in earlier studies, likely because they were conducted before the 2024 ESC Guidelines, which strengthened and clarified recommendations for invasive coronary functional testing for CMD [2]. In addition, the limited availability of this method further restricted its use in clinical research [2,35]. Consequently, former studies may have included heterogeneous patient populations with different underlying etiologies of ANOCA/INOCA. Because the current gold-standard diagnostic methods for CMD are invasive and limited in availability, the implementation of non-invasive and easily accessible imaging tools could potentially offer substantial clinical benefit by enhancing patient comfort and enabling faster and more accurate diagnosis.

Shiromani et al. demonstrated that reduced retinal vessel and perfusion densities in the SCP, assessed by OCTA, were significantly associated with higher odds of CMD among women with INOCA, even after adjustment for major cardiovascular risk factors. These findings suggest that retinal microvascular metrics may serve as potential non-invasive markers of CMD [32]. However, the study was a prospective analysis with a homogeneous cohort; nonetheless, the small sample size, absence of healthy controls, and inclusion of only women limit the generalizability of the findings, which should be validated in larger, mixed-gender populations.

Similar to the findings of Shiromani et al., all other studies using OCTA detected a reduction in VD within the SCP in ANOCA/INOCA population, making it the most extensively investigated vascular parameter across the studies included in this review [29,31,32,34]. The consistent finding of reduced VD in the SCP in studies indicates a reproducible pattern of retinal microvascular changes in patients with ANOCA/INOCA. However, as Eslami et al. found reduced VD in approximately 45% of ANOCA/INOCA patients [34], this suggests limited specificity, which might also result from the lack of detailed evaluation of NOCAD etiology, including CMD.

None of the studies found significant differences in FAZ parameters in OCTA among the study groups. Most former studies found increased FAZ area in patients with CAD compared to controls [9]. Interestingly, Jeremic et al. introduced a novel ellipse-based FAZ analysis that revealed significant differences in FAZ morphology across all CAD severity groups. This relationship was observed only for the left eye. The authors suggest that this asymmetry may be explained by differences in blood flow between the carotid arteries [36]. Zhou et al. found that the left eye shows greater sensitivity to CAD-related changes detected by OCTA than the right eye, suggesting it could be a better indicator for early CAD detection [37]. Among the included studies, the right eye was preferred [26,27,31], the results from both eyes were averaged for further analysis [32], one eye was selected at random [29], or eye selection was not reported [30,33]. Future research should account for possible inter-eye microvascular asymmetry, as single-eye assessment may miss clinically relevant changes in CAD patients.

Choroidal thickness (ChT) measurement has not, to date, entered routine clinical practice. ChT fluctuates during the day and is sensitive to systemic and ocular factors such as age or axial length. This variability, together with the lack of standardization and the inconsistent results across studies investigating ChT in systemic diseases, limits its clinical utility [38,39]. Furthermore, manual caliper measurements are time-consuming and observer-dependent, as the choroid boundary must be identified manually. Kanar et al. evaluated SFCT using SD-OCT and found no significant difference between patients with MVA (ANOCA) and healthy controls [28]. This lack of difference may be related to the relatively small sample size and the reliance on manual measurements.

The assessment of conjunctival circulation using a smartphone combined with a slit-lamp biomicroscope remains a relatively new and unfamiliar technique to most clinicians, and to date, there are no universally standardized measurement protocols or widely established reference norms for bulbar conjunctival microcirculation measurements. The conjunctival microcirculation is directly exposed to environmental and inflammatory factors, which may cause variability and nonspecific changes in vascular parameters [40,41]. In contrast to other imaging modalities, the quality of videos or images is not affected by media opacities; therefore, this technique could be potentially beneficial for individuals where fundus visualization is challenging or impossible due to opaque optical media.

The FWHM method applied to SD-OCT images provides a more objective and reproducible approach for assessing retinal vessel morphology than traditional manual measurements. In this technique, the boundaries of the vessel wall are determined automatically by the algorithm, based on the intensity profile across the vessel [42].

Color fundus photography remains the oldest and most widely used modality for imaging retinal vasculature [43,44]. In Liew et al.’s study, significant differences in retinal vasculature were observed only in venules and only within the female MVA cohort [26]. The authors hypothesized that their findings might reflect better cardiovascular health in women rather than pathological microvascular changes associated with CAD. This interpretation appears plausible and is supported by previous findings showing that narrower retinal arterioles and wider venules are markers of more severe CAD in women [45], and that decreasing arteriolar diameters are associated with an increased risk of CHD in women [46]. Nevertheless, this interpretation remains speculative, as the study did not include a healthy control group for comparison. Moreover, given that this cohort comprised MINOCA patients, these observations should be interpreted as hypothesis-generating and not directly comparable with findings from chronic ANOCA/INOCA cohorts.

Alterations in microvasculature in the eye should not be interpreted in isolation, as multiple cardiovascular risk factors, including hypertension, diabetes, obesity, and dyslipidemia, affect both the retinal and coronary microcirculatory systems similarly [10,11,12]. Retinal venular diameter tends to narrow with age, while wider retinal venular caliber is associated with endothelial dysfunction and inflammation caused by, e.g., smoking, obesity, and higher glycosylated hemoglobin. High blood pressure is associated with narrower arteriolar caliber and wider venular diameter. In contrast to retinal arteriolar caliber, men have wider retinal venular diameters than women [47,48]. Hypertension and diabetes are both associated with reduced retinal vascular density [9]. Smoking, chronic heart failure, and hypertension have been associated with significant choroidal thinning, while choroidal thickness naturally decreases with age. It was found that patients with obstructive CAD have significantly lower choroidal thickness [39], reduced VD (especially in SCP), and enlarged FAZ when compared to healthy controls [9].

In the studies by Kanar, Shiromani, Ren, Liew, and Mailey, multivariable analyses were performed, and the reported associations remained significant even after adjustment for multiple comorbidities [26,28,29,30,32]. Other studies relied on univariate comparisons, group matching, and exclusion criteria rather than multivariable adjustment; therefore, their findings remain more susceptible to residual confounding by prevalent cardiovascular and metabolic comorbidities. The weakest control was observed in Eslami’s study, where high rates of hypertension, diabetes, and hyperlipidemia were present, making it difficult to distinguish ANOCA/INOCA microvascular changes from those driven by underlying systemic disease. Notably, in the Agca et al. study, all patients suffered from obstructive sleep apnea syndrome, a condition associated with reduced retinal perfusion and FAZ alterations. Therefore, the retinal microvascular abnormalities observed in this study cannot be interpreted as arising solely from coronary pathology.

Across all included studies, imaging modalities were non-invasive and enabled rapid image acquisition, but differed substantially in anatomical coverage, processing time, and susceptibility to observer-related variability. Fundus photography and SD-OCT FWHM methods focused on the retinal vessel caliber in Zone B, SD-OCT was applied to choroidal thickness assessment, and the slit-lamp smartphone technique evaluated conjunctival microvasculature, whereas OCTA enabled layer-specific analysis of SCP, DCP, RPC, FD-300, and FAZ. Across all methods, measurements were influenced by patient cooperation, particularly regarding motion and fixation stability.

The most promising imaging modality for microvascular assessment in ANOCA/INOCA is OCTA. This relatively new modality, available since 2016, has been most frequently employed in described studies, likely because it is widely accessible and very simple to operate, even by clinicians without ophthalmologic training. Notably, unlike the other imaging modalities described in this review, OCTA provides fully automated quantification of VD parameters using built-in software algorithms, which substantially reduces observer bias and enhances reproducibility. Unlike other modalities that require manual or semi-manual post-processing or export to external software, OCTA provides immediate quantitative results. The main limitation of OCTA is its small field of view, which may miss peripheral vascular abnormalities. However, this limitation is not unique to OCTA, as vessel assessment in SD-OCT and fundus photography is typically confined to the posterior pole as well [11,49,50,51]. Examples of OCTA imaging could be found in the Agca et al. and Ren et al. studies [29,31] (Table 3).

### 4.1. Future Directions

Across studies evaluating structural, layer-based OCT and OCTA parameters (SFCT, pRNFLT, and RNFL thickness in the RPC) showed inconsistent and limited discriminatory value [28,29]. Among structural biomarkers, retinal ischemic perivascular lesions (RIPLs) are considered the earliest subclinical sign of retinal ischemic infarction. Although RIPLs were not assessed in ANOCA/INOCA patients in the included studies, their evaluation may represent a valuable future direction for detecting subtle ischemic damage in ANOCA/INOCA. RIPLs are defined based on SD-OCT images as a permanent focal thinning of the inner nuclear layer of the retina, resulting from hypoperfusion in the DCP [53,54]. Although nearly half of patients with cardiovascular disease show no RIPLs, the presence of two or more lesions demonstrates high specificity (≈90%). Thus, despite limited sensitivity, RIPLs may serve as a rule-in marker to identify individuals who may benefit from further targeted cardiovascular evaluation [53]. Supportive imaging for RIPLs could be found in a study by Bousquet et al. [55].

To the best of our knowledge, there are still a few other ophthalmological imaging modalities that have not yet been employed for retinal vessel assessment in patients with ANOCA/INOCA. In contrast to fundus photography and the SD-OCT FWHM method, adaptive optics (AO) techniques enable direct visualization of the vessel wall structure due to the very high resolution of approximately 2 µm. Previous studies have demonstrated its capability to detect early microvascular alterations in conditions such as glaucoma, hypertension, and diabetic retinopathy [56,57,58]. In the study by Aschauer et al., patients with coronary heart disease (CHD) exhibited a high prevalence of retinal microvascular abnormalities, including arteriovenous nicking, focal arteriolar narrowing, and microaneurysms, as well as asymmetric vessel wall thickening and hyperreflective intraluminal material. However, no statistically significant differences in quantitative vessel parameters were observed between patients with and without CHD, underscoring the need for larger studies to better establish the role of AO-derived retinal biomarkers in cardiovascular risk assessment [59]. Evaluation of retinal arteriolar morphology with adaptive optics camera (Rtx-1, Imagine Eyes, Orsay, France) was presented by Szewczuk et al. [57]

### 4.2. Limitations

Some limitations are inherited in this systematic review, both from the included studies and from the methodological protocol. Only two publicly available databases (PubMed and Embase) were screened. Updated systematic reviews in the future should screen Web of Science, Google Scholar, and Scopus, too. Moreover, the analysis of ophthalmologic microvascular changes associated with ANOCA/INOCA is a relatively new and emerging concept, and all the included studies were from the last 6 years. Therefore, the number of studies screened and included was relatively small in comparison to systematic reviews on more common topics. The included studies were relatively small, and only one investigation had NOCAD and comparison groups > 100 subjects.

Included articles presented a very heterogeneous methodology and classification system, with only a few fulfilling the exact definition of ESC. In particular, the lack of standardized diagnostic criteria for ANOCA/INOCA across the included studies (e.g., varying stenosis thresholds), together with the fact that only two studies confirmed CMD using invasive coronary functional testing, may have contributed to patient misclassification and influenced the reported associations. Finally, inclusion of a MINOCA cohort—although methodologically valuable—adds clinical heterogeneity. Therefore, this heterogeneity limits the comparability and synthesis of findings across studies. Consequently, updated systematic reviews based on contemporary terminology will be essential in the coming years.

## 5. Conclusions

Ophthalmological diagnostic methods, particularly retinal OCTA, represent promising non-invasive tools for detecting microvascular alterations as a biomarker for evaluating systemic microcirculation abnormalities in ANOCA and INOCA patients. Their advantages include accessibility, rapid acquisition, and quantitative biomarker potential. However, current evidence is limited by small sample sizes, heterogeneity, and a lack of standardized diagnostic thresholds. Further, adequately powered investigations are required to validate these observations, and underexplored modalities such as adaptive optics imaging may provide additional insights into coronary microvascular dysfunction.

## Figures and Tables

**Table 1:** Summary of studies evaluating ophthalmological imaging in patients with non-obstructive coronary artery disease.

Study	Study Type	Blinded Image Analysis	Measurement Method	Imaging Method	NOCAD Subgroup	Comparison Group	Parameters Analyzed	Key Findings	Confounders
**Chronic Coronary Syndromes**									
Xu et al. (2019) [27]	cross-sectional	no	Semi-automated (manual line placement + automated edge detection in ImageJ/FWHM)	SD-OCT (Heidelberg Engineering Inc., Heidelberg, Germany)	MVA (ANOCA/INOCA) * (*n* = 29) (symptomatic, <50% stenosis in primary, <75% in secondary branches)	IHD and healthy controls (*n* = 91) and (*n* = 66) (IHD: MI and/or ≥50% stenosis in primary or ≥75% in secondary branches)	RALD, RAOD, AWT, AVR, VWT, RVOD, RVLD	AVR, RALD, and RAOD differentiated MVA from healthy controls and IHD.	Controlled by statistical adjustment: Age, sex, body mass index, smoking, diabetes, hypertension, dyslipidemia Controlled by exclusion: Eye diseases, past cardiac history, trauma, neurological diseases, liver diseases, kidney diseases, thyroid diseases, pharmacological treatment
Kanar et al. (2021) [28]	cross-sectional	yes	SFCT manual, pRNFLT automated (OCT software)	SD-OCT (RS-3000 Nidek, Gamagori, Japan)	MVA (ANOCA/INOCA) ** (*n* = 32) (symptomatic, ischemia with normal epicardial arteries)	CSFP and healthy controls (*n* = 35) and (*n* = 40) (CSFP: symptomatic, ischemia with angiographically confirmed CSFP)	SFCT, pRNFLT	SFCT and superior and inferior pRNFLT were reduced in CSFP, but not in MVA.	Controlled by statistical adjustment: Hypertension, diabetes, smoking, age, blood pressure, axial length, intraocular pressure, spherical refractive equivalent Controlled by exclusion: Eye diseases (glaucoma, diabetic retinopathy, hypertensive retinopathy, uveitis, high myopia, age-related macular degeneration), ophthalmic surgery, past cardiac history, pharmacological treatment
Ren et al. (2023) [29]	cross-sectional	yes	Automated (OCTA software)	OCTA (RTVue-XR Avanti; Optovue, Fremont, CA, USA)	NOCAD (ANOCA) * (*n* = 65) (symptomatic, 20–50% stenosis on CCTA in all major coronary arteries)	OCAD and healthy controls (*n* = 62) and (*n* = 58) (OCAD: symptomatic, ≥50% stenosis in ≥1 epicardial artery)	Optic disc: VD and RNFL in RPC; macula: VD in SCP and DCP; FD 300	VD reduction in SCP and DCP discriminates NOCAD from OCAD and controls; RNFL inferior quadrant is reduced in OCAD vs. NOCAD.	Controlled by statistical adjustment: Sex, age, body mass index, hypertension, diabetes, dyslipidemia Controlled by exclusion: Eye diseases (retinal vascular occlusion, hypertensive/diabetic retinopathy, glaucoma, macular diseases, ophthalmic surgery, severe refractive error), past cardiac history, vascular diseases
Mailey et al. (CRM) (2023) [30]	cross-sectional	yes	Semi-automated (video stabilization with manual input, automated vessel analysis, and flow quantification)	Slit-lamp + Smartphone Topcon SL-D4 (Topcon Medical Systems Inc., Oakland, NJ, USA) and Apple iPhone 6s (Apple, Inc., Cupertino, CA, USA)	NOCAD (ANOCA) * (*n* = 61) (symptomatic, no obstructive epicardial coronary disease, and FFR ≥ 0.80 in any intermediate lesions)	MI (*n* = 66) (type 1 MI—according to ESC 4th definition)	Vessel diameter, axial and cross-sectional velocity, blood flow rate, wall shear rate	Mean axial and cross-sectional velocity and blood flow rate were significantly lower in the MI vs. NOCAD.	Controlled by statistical adjustment: Age, dyslipidemia, sex, blood pressure, diabetes, smoking, vessel diameter Controlled by exclusion: Conjunctival inflammation, usage of contact lenses, age < 18 years, pregnancy
Mailey et al. (MVR) (2023) [33]	cross-sectional	no	Semi-automated (manual frame selection and vessel classification, automated image analysis, and flow quantification)	Slit-lamp + Smartphone Topcon SL-D4 (Topcon Medical Systems Inc., Oakland, NJ, USA) and Apple iPhone 11 Pro Smartphone (Apple Inc., Cupertino, CA, USA)	INOCA with CMD (*n* = 43) (symptomatic, no stenosis > 50%, FFR ≥ 0.80, IMR ≥ 25 or CFR < 2.0)	Controls (*n* = 68) (symptomatic, no stenosis > 50%, FFR ≥ 0.80, IMR < 25, CFR ≥ 2.0)	Vessel diameter, axial and cross-sectional velocity, blood flow rate, wall shear rate, wall shear stress	Axial and cross-sectional velocity, wall shear and stress rate, and blood flow rate were reduced in CMD.	Controlled by statistical adjustment: Sex, age, body mass index, smoking, hypertension, diabetes, dyslipidemia, pharmacological treatment Controlled by exclusion: Conjunctival inflammation, usage of contact lenses, past cardiac history, trauma, age < 18 years, pregnancy
Agca et al. (2023) [31]	cross-sectional	partial (cardiologists blinded)	Automated (OCTA software)	OCTA (RTVue XR Avanti; Optovue Inc., Fremont, CA, USA)	INOCA with OSAS (*n* = 94) (symptomatic, <50% stenosis or FFR > 0.80)	OCAD with OSAS and healthy controls (*n* = 29) and (*n* = 62) (OCAD: symptomatic, ≥50% stenosis in ≥1 major coronary artery)	VD in SCP, DCP, RPC, FAZ	Reduced VD in SCP, DCP, RPC in OCAD and INOCA vs. controls; VD was higher in women with INOCA vs. OCAD.	Controlled by statistical adjustment: Sex, age, body mass index, smoking, blood pressure, dyslipidemia, diabetes Controlled by exclusion: Central sleep apnea, obesity hypoventilation syndrome, insomnia, past and acute cardiac history, hypertensive/diabetic retinopathy, intraocular inflammatory diseases, ocular surgeries other than cataract, conditions obscuring macula or fundus, previous ophthalmologic disease, ocular trauma or tumor, amblyopia, best-corrected sharp visual acuity worse than 20/20, cylindrical > 2 diopters and/or 4 diopters spherical refractive error, intraocular pressure > 21 mmHg, poor image quality
Shiromani et al. (2025) [32]	cross-sectional	no	Automated (OCTA software: Cirrus Review Software V11.1.0.32456)	OCTA (Carl Zeiss Meditec Inc., Dublin, CA, USA)	INOCA with CMD (*n* = 11) vs. INOCA without CMD (*n* = 7) (symptomatic women, CMD: CFR < 2.5)	—	VD, perfusion density in SCP, FAZ	Reduced vessel and perfusion density in INOCA with CMD.	Controlled by statistical adjustment: Sex, age, body mass index, diabetes, blood pressure, dyslipidemia, smoking, pharmacological treatment Controlled by exclusion: Past and acute cardiac history, eye diseases (ocular media opacity preventing acquisition of high-quality retinal images), liver diseases, kidney diseases
Eslami et al. (2021) [34]	cross-sectional	yes	Automated (OCTA software)	OCTA (no information)	Normal angiographic patients (ANOCA/INOCA) * (*n* = 60) (symptomatic, no ‘endoluminal irregularities’ or stenosis < 20% or mild CAD 20–50% abnormal non-invasive tests (ECG/echocardiography, exercise tests)	—	VD in SCP, DCP, FAZ	45% of normal angiographic patients had abnormal VD in SCP.	Measured confounders: Age, diabetes, hypertension, dyslipidemia, smoking, sex, electrocardiogram Controlled by exclusion: Slow-flow phenomenon, coronary ectasia, missing data
**Acute Coronary Syndromes**									
Liew et al. (2019) [26]	cross-sectional	yes	Semi-automated (computer-assisted method with manual verification)	Fundus photography Canon 60° fundus camera (CF-60DSi, Canon Inc., Tokyo, Japan) and digital camera (1DSmkIII, Canon Inc., Tokyo, Japan).	MVA (MINOCA) ** (*n* = 139) (symptomatic, <25% stenosis in all epicardial arteries)	CAD (*n* = 776) (≥25% stenosis in ≥1 epicardial artery)	Retinal arteriolar and venular calibers	Women with narrower venules were 3× more likely to have MVA.	Controlled by statistical adjustment: Age, ethnicity, sex, mean arterial pressure, body mass index, fellow vessel caliber, diabetes, hypertension, smoking, stroke Other measured confounders: Total cholesterol, HbA1c, fasting blood glucose, past cardiac history

* The study groups were classified according to the new nomenclature, according to the 2024 ESC Guidelines for the management of chronic coronary syndromes. ** Liew et al. [26] enrolled MINOCA patients. Given the distinct and heterogeneous pathophysiology of MINOCA, results from this study are presented separately and should not be directly extrapolated to chronic ANOCA/INOCA. ANOCA, angina with non-obstructive coronary arteries; AVR, arteriovenous ratio; AWT, arterial wall thickness; CAD, coronary artery disease; CCTA, coronary computed tomography angiography; CFR, coronary flow reserve; CMD, coronary microvascular dysfunction; CSFP, coronary slow-flow phenomenon; DCP, deep capillary plexus; ECG, electrocardiogram; ESC, European Society of Cardiology; FAZ, foveal avascular zone; FD, foveal density; FFR, fractional flow reserve; HbA1c, hemoglobin A1c; IHD, ischemic heart disease; IMR, Index of Microcirculatory Resistance; INOCA, ischemia with non-obstructive coronary arteries; MI, myocardial infarction; MINOCA, myocardial infarction with non-obstructive coronary arteries; MVA microvascular angina; NOCAD, non-obstructive coronary artery disease; OCTA, optical coherence tomography angiography; OCAD, obstructive coronary artery disease; OSAS, obstructive sleep apnea syndrome; pRNFLT, peripapillary retinal nerve fiber layer thickness; RALD, retinal arterial lumen diameter; RAOD, retinal arterial outer diameter; RNFL, retinal nerve fiber layer; RPC, radial peripapillary capillaries; RVLD, retinal venular lumen diameter; RVOD, retinal venular outer diameter; SCP, superficial capillary plexus; SD-OCT, spectral-domain optical coherence tomography; SFCT, subfoveal choroidal thickness; VD, vessel density; VWT, venous wall thickness.

**Table 2:** Quality of the included studies.

Study	Were the Criteria for Inclusion in the Sample Clearly Defined?	Were the Study Subjects and the Setting Described in Detail?	Was the Exposure Measured in a Valid and Reliable Way?	Were Objective, Standard Criteria Used for Measurement of the Condition?	Were Confounding Factors Identified?	Were Strategies to Deal with Confounding Factors Stated?	Were the Outcomes Measured in a Valid and Reliable Way?	Was Appropriate Statistical Analysis Used?
Liew et al. (2019) [26]	Yes	Yes	Yes	Yes	Yes	Yes	Yes	Yes
Xu et al. (2019) [27]	Yes	Yes	Yes	Yes	Yes	Unclear	Yes	Yes
Kanar et al. (2021) [28]	Yes	Yes	Yes	Yes	Yes	Yes	Yes	Yes
Ren et al. (2023) [29]	Yes	Yes	Yes	Yes	Yes	Yes	Yes	Yes
Mailey et al. (CRM) (2023) [30]	Yes	Yes	Yes	Yes	Yes	Yes	Yes	Yes
Mailey et al. (MVR) (2023) [33]	Yes	Yes	Yes	Yes	Yes	Yes	Yes	Yes
Agca et al. (2023) [31]	Yes	Yes	Yes	Yes	Yes	Unclear	Yes	Yes
Shiromani et al. (2025) [32]	Yes	Yes	Yes	Yes	Yes	Yes	Yes	Yes
Eslami et al. (2021) [34]	Yes	Yes	Yes	Yes	Yes	Unclear	Yes	Yes

**Table 3:** Overview of ocular imaging modalities for retinal microcirculation and their potential relevance to coronary microvascular dysfunction.

Ocular Imaging Modality	Key Features	Relevance to Coronary Microvascular Dysfunction
Color Fundus Photography	Two-dimensional color retinal images enabling assessment of large vessels and retinopathy signs (e.g., microaneurysms, hemorrhages, hard exudates, venous beading, neovascularization) and measurement of retinal vessel caliber, typically in Zone B [10,47,48].	Allows qualitative and quantitative analysis (e.g., vessel caliber, tortuosity, fractal dimension), which are associated with general cardiovascular risk factors and outcomes. Useful for population-based studies and basic risk assessment [8,14,52].
Spectral-Domain Optical Coherence Tomography	High-resolution, cross-sectional imaging of retinal and choroidal layers. Assesses structural features and thickness [12].	Reduced thickness of the retina, choroid, and RNFL has been reported in patients with CAD [9].
Optical Coherence Tomography Angiography	A functional extension of OCT that visualizes blood flow signals without contrast injection. Provides depth-resolved, high-resolution images of microvascular networks, with automated quantification of vessel density and perfusion density in SCP, DCP, and RPC, as well as FAZ metrics [50,51].	This is a key non-invasive modality for the assessment of retinal microvascular alterations relevant to CMD. It quantifies microvascular changes associated with CAD, such as decreased vessel density and reduced vessel length density [9].

Abbreviations: CAD, coronary artery disease; CMD, coronary microvascular dysfunction; DCP, deep capillary plexus; OCT, optical coherence tomography; SCP, superficial capillary plexus; RNFL, retinal nerve fiber layer; RPC, radial peripapillary capillaries; FAZ, foveal avascular zone.

## Data Availability

The raw data supporting the conclusions will be made available on a reasonable request to the corresponding author.

## Supplementary Materials

The following supporting information can be downloaded at https://www.mdpi.com/article/10.3390/jcm15041344/s1. Table S1: PRISMA checklist.

## Abbreviations

The following abbreviations are used in this manuscript:

ANOCA	Angina with non-obstructive coronary arteries
AO	Adaptive optics
AWT	Arterial wall thickness
AVR	Arteriovenous ratio
CAD	Coronary artery disease
CCTA	Coronary computed tomography angiography
CHD	Coronary heart disease
CHT	Choroidal thickness
CMD	Coronary microvascular dysfunction
CSFP	Coronary slow-flow phenomenon
DCP	Deep capillary plexus
ESC	European Society of Cardiology
FAZ	Foveal avascular zone
FWHM	Full-width half-maximum
ICA	Invasive coronary angiography
INOCA	Ischemia with non-obstructive coronary arteries
MINOCA	Myocardial infarction with non-obstructive coronary arteries
MVA	Microvascular angina
NOCAD	Non-obstructive coronary artery disease
OCAD	Obstructive coronary artery disease
OCT	Optical coherence tomography
OCTA	Optical coherence tomography angiography
OSAS	Obstructive sleep apnea syndrome
pRNFLT	Peripapillary retinal nerve fiber layer thickness
RALD	Retinal arterial lumen diameter
RAOD	Retinal arterial outer diameter
RF-CL	Risk-factor-weighted clinical likelihood
RIPLs	Retinal ischemic perivascular lesions
RNFL	Retinal nerve fiber layer
RPC	Radial peripapillary capillaries
RVLD	Retinal venular lumen diameter
RVOD	Retinal venular outer diameter
SCP	Superficial capillary plexus
SD-OCT	Spectral domain optical coherence tomography
SFCT	Subfoveal choroidal thickness
VD	Vessel density
VSA	Vasospastic angina
VWT	Venous wall thickness
